# The presence of *Enterococcus faecalis* in saliva as a risk factor for endodontic infection

**DOI:** 10.3389/fcimb.2023.1061645

**Published:** 2023-04-06

**Authors:** Carlo Gaeta, Crystal Marruganti, Islam A. A. Ali, Andrea Fabbro, David Pinzauti, Francesco Santoro, Prasanna Neelakantan, Gianni Pozzi, Simone Grandini

**Affiliations:** ^1^ Unit of Periodontology, Endodontic and Restorative Dentistry, Department of Medical Biotechnology, University of Siena, Siena, Italy; ^2^ Department of Endodontics, Faculty of Dentistry, Mansoura University, Mansoura, Egypt; ^3^ Faculty of Dentistry, The University of Hong Kong, Sai Ying Pun, Hong Kong, Hong Kong SAR, China

**Keywords:** apical periodontitis, endodontic infections, *enterococcus faecalis*, phylogenetic analysis, risk factor, saliva, whole-genome sequencing (WGS)

## Abstract

**Aim:**

The aim of the present study was to investigate and correlate the prevalence of *Enterococcus faecalis* in saliva and in root canals with different pulpal and periapical conditions.

**Methodology:**

Sixty-seven patients were divided into five groups based on pulpal and periapical tissue status: healthy vital teeth (HVT, n=7), healthy treated teeth without lesion (HTT, n=9), irreversible pulpitis (IP, n=13), necrosis (N, n=18), and post-treatment apical periodontitis (PTAP, n=20). Saliva, rubber dam, sterility control and pre-treatment root canal samples were collected and microbiologically processed by culture method. The phylogenetic relationship of *E. faecalis* isolates collected from root canals and saliva were investigated by whole genome sequencing. Fisher’s exact test was used to correlate the presence of *E. faecalis* in root canals or saliva with clinical and/or radiographic findings. Linear/logistic regression analyses were performed to establish the relationship between the presence of *E. faecalis* in root canals, saliva, and the status of periapical tissues.

**Results:**

*E. faecalis* was found in 18 root canal and saliva samples. *E. faecalis* root canal isolates were recovered with the highest frequency from post-treatment apical periodontitis. The occurrence of *E. faecalis* in saliva was strongly associated with its detection in the root canals (*P* < 0.001). The pretreatment presence of *E. faecalis* in root canals was associated with significantly higher odds of having periapical lesions (OR=11.03; 95% CI, 1.27-95.70; *p* < 0.05). Saliva and root canal isolates from the same patient were highly correlated at the phylogenetic level (Jaccard index >0.95).

**Conclusion:**

This pilot study confirms the role of *E. faecalis* in developing peri-radicular lesions in secondary endodontic infections and suggests that saliva could be the main source of infection. Further studies are needed to investigate the exact origin of this bacteria and its true role in the pathogenesis of secondary/persistent endodontic infections.

## Introduction

The role of bacteria in the initiation and progression of apical periodontitis has been widely demonstrated (Kakehashi et al., 1965). Bacteria can invade the root canal system *via* different pathways such as carious, periodontal lesions and cracks (Siqueira and Rôças, 2009). Microbial profiling of endodontic infections revealed compositionally unspecific, yet differentially abundant microbiota depending on clinical diagnosis (Manoil et al., 2020). While primary infections are caused by microorganisms that initially invade and colonize necrotic root canals, secondary and persistent infections are caused by microorganisms that enter root canals as a result of professional intervention or survive the chemo-mechanical debridement and persist within the root canal environment (Wong et al., 2021). *Enterococcus faecalis* is a facultative anaerobic gram-positive bacterium which has been frequently recovered from secondary/persistent endodontic infections (Rôças et al., 2004; Sedgley et al., 2006b; Bouillaguet et al., 2018). The contribution of *E. faecalis* to endodontic treatment failures is attributed to its ability to withstand nutrient scarcity encountered in root-filled teeth (Evans et al., 2002; Stuart et al., 2006) and tolerance to antimicrobials employed during endodontic treatment (Ali et al., 2020a; Ali et al., 2020b). The ability of *E. faecalis* to form dense biofilms on root canal walls, by a biofilm-associated pili (Ebp) and its collagen-binding protein (Ace), make this microorganism able to invade dentinal tubules and root canal complexities and contributing to be recalcitrant to endodontic disinfectants and intracanal dressings (Zhang et al., 2015; Hahn and Hanford, 2021; Momenijavid et al., 2022). Also many other virulence factors and its predisposition to be resistant to some antibiotics contribute to persistence and recovery of *E. faecalis* from endodontic failures (Aas et al., 2005; Gomes et al., 2021). Given that endodontic microbiota is derived from oral microbiota under the influence of specific ecological conditions of root canal environment (Wong et al., 2021), and despite the recovery of *E. faecalis* from root-filled teeth with post-treatment diseases, *E. faecalis* is not a typical member of commensal oral microbiota (Aas et al., 2005). It is less likely that *E. faecalis* occurs in advanced carious lesions, and primary endodontic infections (Martin et al., 2002). Therefore, the origin of *E. faecalis* recovered from root canals has been questioned, and its association with their respective counterparts in saliva was studied (Zehnder and Guggenheim, 2009). A significant association was found between the presence of *E. faecalis* in saliva and root canals with post-treatment apical periodontitis (Wang et al., 2012). Similar genotype was detected in *E. faecalis* isolated from saliva and endodontically treated teeth (Delboni et al., 2017), while different genetic profiles were observed in salivary and root canals strains from the same patient (Zhu et al., 2010). Therefore, the origin of *E. faecalis* in endodontic treatment failures was proposed to be exogenous (Vidana et al., 2011). With such contradictory findings, the relationship between *E. faecalis* in saliva and root canals remains unsolved and additional evidence is warranted. Therefore, the aim of this study was to determine the prevalence of *E. faecalis* in root canals and saliva and to investigate whether its presence could influence the presence and dimension of periapical lesions.

## Materials and methods

### Study design

The present cohort study is reported following the Strengthening the Reporting of Observational studies in Epidemiology (STROBE) guidelines for cohort studies (Von Elm et al., 2008). The research protocol was approved by the local Ethics Committee (protocol number: 18202/2020) and was registered on Clinicaltrials.gov (NCT04637659).

### Setting and participants

Sixty-seven patients were sequentially recruited among those attending the Unit of Endodontology and Restorative dentistry, School of Dentistry, University of Siena between July 2020 and November 2020 according to the following eligibility criteria:

- need for a root canal treatment or retreatment with previous therapy aging for at least five years.
- ability and willingness to give informed consent.
The exclusion criteria were:
- presence of periodontitis (Tonetti et al., 2018).
- impossibility to isolate the operating field.
- retreatment cases with missing or calcified canals, perforation and separated endodontic instruments in which was impossible to reach the apex.
- administration of antibiotics within the last 3 months.
- patients with diabetes, rheumatoid arthritis, and inflammatory bowel diseases.

The cohort of patients included in the present study was defined once all participants read and signed a written informed consent, according to the Declaration of Helsinki.

### Variables

#### Clinical and radiographic assessment

For each participant, demographic characteristics (age, gender) as well as medical and dental history were collected. Tooth position (anterior/posterior) and type of coronal restoration (direct/indirect) were recorded during the clinical examination; The quality of each restoration was defined as proper or improper, according to the “Modified USPHS ‘‘ criteria (Bayne and Schmalz, 2005). A standardized periapical intraoral radiograph was performed to evaluate the status through the Periapical Index (PAI) score (Orstavik et al., 1986). Afterwards, the included teeth were categorized into five groups according to their pulpal and periapical status as determined by clinical and radiographic findings: (i) healthy vital tooth (HVT) group was represented by a clinical situation in which endodontic treatment is needed for prosthetic reasons despite the pulp not showing any sign of inflammation; (ii) healthy treated tooth (HTT) group included teeth in which the pre-existing endodontic filling material was exposed to oral cavity with no sign of periapical lesion; (iii) irreversible pulpitis (IP) diagnosed by sharp spontaneous pain and tenderness to percussion or pain exacerbated by lying down or cold test (Levin et al., 2009); (iv) pulp necrosis (N) group belonged to untreated teeth, negative to cold test, with and without apical periodontitis; post-treatment apical periodontitis (PTAP).

#### Sampling and clinical procedures

Root canal and saliva samples were collected as previously described (Sedgley et al., 2006a). Before isolation with the rubber dam, saliva samples from the floor of the mouth, dorsum of the tongue and the crown of the affected tooth were collected for each patient using three sterile ISO size 40 paper points (Dentsply-Maillefer, Ballaigues, Switzerland). The paper points were resuspended in 100 μl of PBS/10% glycerol and stored at -70°C until analysis. Plaque around the affected tooth was removed using scalers and the surfaces were brushed with pumice. Teeth were isolated with a rubber dam and disinfected with 30% hydrogen peroxide and 5.25% sodium hypochlorite (NaOCl), which is inactivated by sodium thiosulphate 5%. As a sterility control, three sterile paper points (Size 40) were rubbed on the crown of the tooth and on the surrounding areas. After access preparation, root canal patency was achieved with minimal instrumentation and without using hypochlorite irrigant. In case of retreatment, coronal gutta percha was removed by sterile Gates Glidden drills size 2 & 3 (Dentsply-Maillefer, Ballaigues, Switzerland), while the middle and apical gutta percha were removed with endodontic files without a chemical solvent. Irrigation was performed with sterile saline to remove any residual material before the collection of the intracanal sample. Once the working length was established, the pre-treatment sample was collected using ISO size 10 K-file (Dentsply-Maillefer, Ballaigues, Switzerland). An additional pretreatment sampling was performed by introducing two sterile paper points (ISO size 15) into the full working length kept for at least 60 seconds. The sample was then transferred to PBS/10% glycerol solution. When the canal was dry, a sterile paper point moistened with sterile saline was used to acquire the sample. In multi-rooted teeth, a single root canal was chosen, based on the presence of periapical radiolucency and/or exudation.

### Laboratory assessment

#### Isolation and identification of *Enterococci*

Ten μl of PBS/10% glycerol from each sample were plated on Brain Heart Infusion (BHI) agar containing 5% horse blood. The plates were incubated in 5% CO_2_ at 37°C for 48 hours and monitored daily for the presence of microbial growth. Putative enterococcal colonies were isolated on BHI agar/blood and identified with a latex agglutination test (Oxoid™ Streptococcal Grouping Kit, Thermo Fisher, Hampshire, United Kingdom). Group D colonies were then identified on a MALDI Biotyper (Bruker Daltonics, Bremen, Germany) and by ribosomal RNA operon sequencing (Cuscó et al., 2018). Colonies identified as *E. faecalis* were frozen at -70°C in BHI/10% glycerol.

#### High molecular weight DNA extraction

*E. faecalis* strains were streak plated on BHI agar/blood, incubated overnight at 37°C and checked for purity. About ten single colonies were inoculated in BHI broth and the starter cultures of exponentially growing bacteria (OD_590_ of 0.3-0.4) were frozen at -70°C with 10% glycerol. Bacteria were inoculated 1:50 (vol:vol) from starter cultures in 10 ml of BHI broth and incubated at 37° C until an OD_590_ of 1.0 was reached. Samples were then centrifuged at 6600 x *g* for 5 minutes. Bacterial pellets were washed with 10 ml of sterile 1X TE buffer (Tris 10 mM-EDTA 1 mM) and resuspended in 7.5 ml of Raffinose buffer (50 mM Tris pH 8, 5 mM EDTA, 20% Raffinose). DNA extraction was carried out as described previously (Pinzauti et al., 2022). The DNA pellet was resuspended in 100 μl of saline. Genomic DNA was quantified using a Qubit 2.0 fluorometer (Invitrogen, Whaltan, Massachusetts, USA) and a NanoPhotometer device (Implen, Westlake Village, USA) before molecular analysis and whole genome sequencing.

#### Sequencing and bioinformatic analysis

Whole genome sequencing (WGS) was performed employing Oxford Nanopore technology. Following manufacturers’ instruction, the sequencing library was prepared using a ligation sequencing kit (SQK-LSK108) and barcode expansion kits (EXP-NBD104/114) for sample multiplexing. The sequencing run was performed on the GridION x5 platform (Oxford Nanopore Technologies). Nanopore reads were filtered using the tool Filtlong (v. 0.2.0) (https://github.com/rrwick/Filtlong) removing reads shorter than 1,000 bases (–min_length 1000) and getting rid of the 5% worst (low quality) reads (–keep_percentage 95). Samples were also sequenced with Illumina technology at MicrobesNG (Birmingham, UK) (https://microbesng.com/) which performed library preparation and sequencing of paired end 250 bp reads on a HiSeq2500. Raw Illumina reads were quality checked at MicrobesNG: reads were trimmed using Trimmomatic (v. 0.30) (Bolger et al., 2014) and analyzed with FastQC (v. 0.11.5) (http://www.bioinformatics.babraham.ac.uk/projects/fastqc).

High quality complete genomes were *de novo* assembled using Unicycler (v 0.4.7) (Wick et al., 2017), with both Nanopore and Illumina reads as an input. Phylogenetic relationships among sequenced genomes were explored using PopPUNK (v. 2.4.0) using the ‘fit-model lineage’ parameter for data fitting (Lees et al., 2019). PopPUNK exploits the Jaccard index (J) to establish the similarity between k-mer data sets (oligonucleotide sequences of k length) of two genome sequences (0<J<1, with J=1 describing two genome sequences sharing the same k -mers) (De Giorgi et al., 2022).

### Power analysis

The detection rate of *E. faecalis* in culture medium was reported to be 2% and 71% in primary and secondary endodontic infections respectively (Guo et al., 2011). Therefore, setting the level of significance at alpha=0.05, the power of the study resulted to be above 90%.

### Statistical analysis

All analyses were performed using a statistical software (STATA BE, version 17.1, StataCorp LP, Texas, USA), setting the level of significance at 5%. Continuous variables were expressed as Mean (SD), while categorical variables were expressed as number of observations (percentage - %). Fisher’s exact test was used to investigate the association between clinical and microbiological variables. Simple linear/logistic multilevel regression models were built in order to evaluate the association between *E. faecalis* presence in the canal before treatment/*E. faecalis* presence in saliva and tooth vitality, presence of periapical lesion and PAI score, respectively. Multiple multilevel regression models were obtained by adjusting the crude estimates for confounders (i.e. proper/improper restoration, type of restoration, tooth position).

## Results

### Participants and samples

Sixty-seven patients (36 males and 31 females), aged from 26 to 90 (mean ± SD = 56 ± 1.67), were included in the study. A total of 79 teeth in the recruited patients were sampled. Eleven samples were discarded due to sampling or laboratory errors, and one saliva sample was repeated in the same patient after 4 months after endodontic therapy. Therefore, a total of 67 teeth were included in the final analysis. *E. faecalis* was recovered from 11 (16.42%) and 7 (10.45%) root canal and saliva samples, respectively. The highest frequency was from the PTAP group (30%), followed by N (22.2%) and HTT (16.6%) groups. *E. faecalis* was not detected in IP and HVT groups. Descriptive statistics of patients’ characteristics, and clinical and microbiological assessments are shown in **Table 1**.

**Table 1:** Descriptive statistics of patients’ characteristics.

Variable	Mean ± SD/Proportion (%)
Age	55.69 ± 1.67
Gender	
* Females*	31 (45.56%)
* Male*	36 (54.44%)
Groups	
* HVT*	7 (10.45%)
* HTT*	9 (13.43%)
* IP*	13 (19.40%)
* N*	18 (26.87%)
* PTAP*	20 (29.85%)
Lesion	
* Present*	49 (73.13%)
* Absent*	18 (26.87%)
PAI score	
* 0*	18 (26.87%)
* 1*	16 (23.88%)
* 2*	7 (10.45%)
* 3*	20 (29.85%)
* 4*	4 (5.97%)
* 5*	2 (2.99%)
Position	
* Anterior*	26 (38.81%)
* Posterior*	41 (61.19%)
Restoration type	
* Indirect*	46 (68.66%)
* Direct*	21 (31.34%)
Saliva	
* Present*	7 (10.45%)
* Absent*	60 (89.55%)
Canal pre-treatment	
* Present*	11 (16.42%)
* Absent*	56 (83.58%)

SD, standard deviation; HVT, healthy vital tooth; HTT, healthy treated tooth; IP, irreversible pulpitis; N, necrotic tooth; PTAP, post-treatment apical periodontitis; PAI score, periapical index score. Saliva +, proportion of saliva samples positive for E. faecalis; Canal pre-treatment, proportion of samples in the canal before treatment positive for E. faecalis.

### Outcome data

#### Clinical and microbiological variables

Results of the association between clinical and microbiological variables are shown in **Table 2**. The presence of *E. faecalis* in root canals before the endodontic treatment (pre-treatment) was significantly associated with the presence in saliva (*p*<0.001) and with the presence of radiologically evident periapical lesions (*p<*0.05). The tooth position, type and quality of coronal restorations were not significantly associated with the presence of *E. faecalis* in any of the samples.

**Table 2:** Inferential statistics and Fisher’s test values.

Variable/*E. faecalis* in sample	Saliva (yes/no)	p value	Pre-treatment (yes/no)	p value
Group				
* HVT (n=7)*	0/7	0.103	0/7	0.139
* HTT (n=9)*	0/9		2/7	
* IP (n=13)*	0/13		0/13	
* N (n=18)*	5/13		3/15	
* PTAP (n=20)*	2/18		6/14	
Lesion				
* Present (n=49)*	6/43	0.665	11/49	0.023*
* Absent (n=18)*	1/17		0/18	
PAI score				
* 0 (n=18)*	1/17	0.071	0/18	0.030*
* 1 (n=16)*	0/16		3/13	
* 2 (n=7)*	0/7		0/7	
* 3 (n=20)*	4/16		6/14	
* 4 (n=4)*	1/3		2/2	
* 5 (n=2)*	1/1		0/2	
Quality of restoration				
* Proper (n=10)*	1/9	0.132	0/10	0.195
* Improper (n=57)*	6/51		11/57	
Type of restoration				
* Direct (n=46)*	1/45	0.721	7/39	0.730
* Indirect (n=21)*	6/15		4/17	
Position				
* Anterior (n=26)*	4/22	0.257	5/21	0.738
* Posterior (n=41)*	3/38		6/35	
Pre-treatment sample				
* E. faecalis present*	5/7	0.001**	—	—
* E. faecalis absent*	6/60		—	

HVT, healthy vital tooth; HTT, Healthy treated teeth; IP, irreversible pulpitis; N, necrotic tooth; PTAP, post-treatment apical periodontitis; Dash symbol, not measured; PAI score, periapical index score. The saliva and pre-treatment columns report the number of saliva (n=67) and pre-treatment samples (n=67) positive/negative for the presence of E. faecalis in the categories described within each line. The p value columns report the result of Fisher’s exact test used to investigate the association between variables reported in lines with those reported in columns. Bold value denote statistical significance (*p<0.05,**p<0.001).

#### Linear/logistic regression analyses

The combined effect of the variables that were related to the presence of *E. faecalis* in pre-treatment samples were investigated using a logistic regression model (**Table 3**). The presence of *E. faecalis* in root canal samples before the treatment significantly increased the odds of having a secondary endodontic infections (OR=2.94; 95% CI [1.47, 11.59]; *p*<0.05) while its presence in saliva was associated with higher odds of identifying *E. faecalis* in root canals (OR=3.70; 95% CI [1.031, 19.229]; *p*<0.05) and to develop a secondary/persistent infection (OR=3.07; 95% CI [1.67, 6.88]; *p*<0.05). The presence of *Enterococcus faecalis* in root canals significantly increased the odds of periapical lesion (OR=11.03; 95% CI [1.273, 95.704]; *p*<0.05). However, this was not the case when *E. faecalis* was identified in saliva (OR=1.97; 95% CI [0.333, 11.674]; *p*<0.454). Finally, the presence of *E. faecalis* in pretreatment samples increases the odds of a higher PAI index score (MD=1.031; 95% CI[0.091, 1.971]; *p*<0.05).

**Table 3:** Linear/logistic regression analyses for the association between *E.faecalis* in the root canal/saliva and PAI score with clinical variables, respectively.

Variable	Presence of *Enterococcus faecalis* in root canal before treatment										
	N	Crude ORs		95% CI		*p*-value^*^	N	Adjusted^†^ ORs	95% CI		*p*-value^*^
				Lower	Upper				Lower	Upper	
Secondary infection groups	67	3.6		1.5	13.63.1	0.049*	57	2.95	1.5	11.6	0.023*
Presence of periapical lesion	67	9.3		77.6	12.4	0.039*	57	11.0	1.2	95.7	0.029*
**Presence of *Enterococcus faecalis* in saliva**											
Secondary infection groups	67	0.9		0.16	5.26	0.938	67	3.07	1.67	6.88	0.040*
Presence of periapical lesion	67	1.9		0.3	10.6	0.460	67	1.9	0.3	11.6	0.454
**PAI score**											
	**N**	**Crude ORs**		**95% CI**		*p*-value^*^	**N**	**Adjusted** ^†^ **ORs**	**95% CI**		*p*-value^*^
				**Lower**	**Upper**				**Lower**	**Upper**	
*Enterococcus faecalis* in pre-treatment samples	67	1.08		0.1	2.0	0.022*	67	1.03	0.1	1.9	0.032*

Abbreviations: ORs, odds ratios; CI, confidence interval; secondary infection group (PTAP,HTT).

Bold value denote statistical significance at^*^ *p*<0.05, ^†^Adjusted for tooth position, type and proper/improper restoration.

#### Whole genome sequencing and phylogenetic relationships of *E. faecalis* isolates

**Figure 1:**
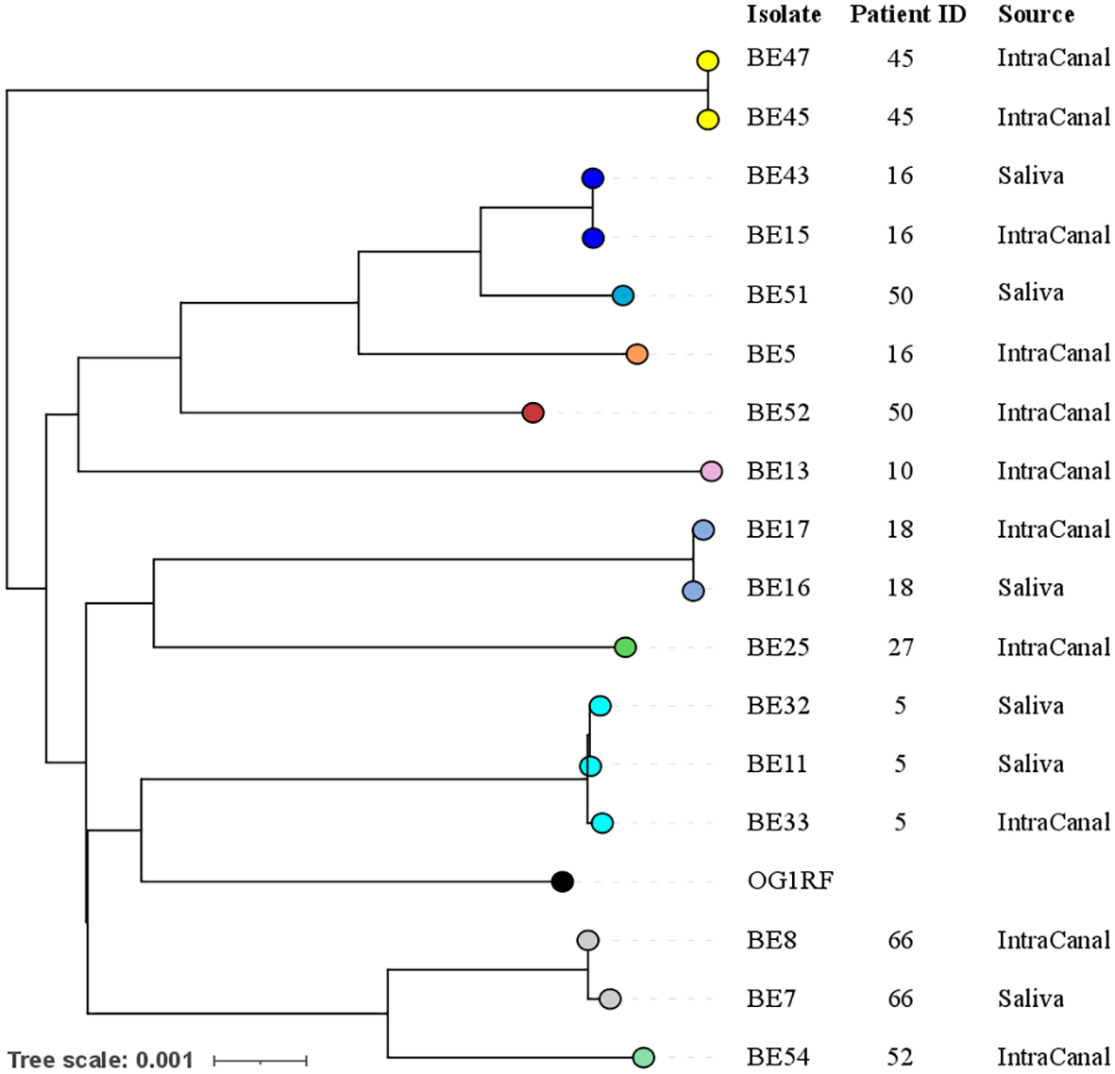
Phylogenetic relationships among *E. faecalis* isolates. Different colors indicate different genomic clusters: ten population clusters were identified using core genome sequences, 5 clusters contain a single genome, while another 5 contain either 2 or 3 genomes, notably all coming from the same patient. The phylogenetic tree was generated based on whole genome sequences with branch lengths indicating the number of nucleotide substitutions per site (scale bar). Patient ID and source of the sample are indicated on the right. The genome of the reference laboratory strain OG1RF was used as an outgroup.

Seventeen out of 18 *E. faecalis* strains were sequenced using both Illumina and Nanopore technologies (n= 17) or only with Nanopore (n=14). One strain was not vital and could not be sequenced. Genomes were assembled and the whole genome sequences were used to investigate the phylogenetic relationships among different isolates with PopPUNK (**Figure 1**). Core genome analysis and whole genome analysis identified five major clusters. Analysis of phylogenetic distances among isolates using the Jaccard index (J) with different k-mer lengths indicated that saliva and root canal isolates of *E. faecalis* retrieved from the same patient, BE15 and BE43, BE16 and BE17, BE7 and BE8, BE32 and BE33 were highly correlated (J>0.95). Moreover, the strains BE11 and BE32 were recovered from the saliva of the same subjects at 2 different visits 4 months apart, and share a J index of 0.987 (k=29). The strains BE5 and BE52 share a J index of 0.8 although they were recovered from root canals of different subjects.

## Discussion

The present study aimed to assess the prevalence and correlation between *E. faecalis* isolates from root canals, with different pulpal and periapical conditions, and saliva to better understand the origin of *E. faecalis* in endodontic infections. This study analyzed the association of *E. faecalis* presence in root canals before treatment with (i) the status of periapical tissues, and (ii) clinical characteristics such as type, quality and location of restorations. Previous studies investigated the prevalence of *E. faecalis* in failed endodontic treatment and persistent infections (Zhu et al., 2010; Wang et al., 2012; Delboni et al., 2017), and recovered *E. faecalis* from primary endodontic infections (Rôças et al., 2004; Sedgley et al., 2006a; Guo et al., 2011). This study included clinical conditions ranging from healthy pulp to teeth with post-treatment apical periodontitis, classifying each condition into primary or secondary/persistent endodontic infection groups as previously established (Delboni et al., 2017). A cultural approach was used to isolate *E. faecalis* from saliva and endodontic samples, this allowed to recover strains for further molecular characterization and to avoid PCR-based techniques, which could be influenced by contamination and by the presence of extracellular DNA or DNA from dead bacterial cells (Siqueira, 2002; Gomes et al., 2015). Recently, Next Generation Sequencing (NGS)-based studies revealed an unspecific composition of endodontic microbiota (Wong et al., 2021), and challenged the role of *E. faecalis* in the etiology of persistent/secondary root canal infections (Rogers et al., 2010; Brundin et al., 2014) even if a recent 16S rRNA amplicon sequencing study detected high abundance of *E. faecalis* OTUs in secondary apical periodontitis (Bouillaguet et al., 2018). *E. faecalis* was not identified in root canals with healthy vital pulp or irreversible pulpitis, coherently with the reported absence of *E. faecalis* in carious lesions close to the pulp (Martin et al., 2002). On the other hand, a more recent NGS-based study identified the genus *Enterococcus* in the microbiome of root canals with irreversible pulpitis, albeit at a very low relative abundance (Siqueira et al., 2016). According to our study, the prevalence of *E. faecalis* in necrotic root canals was 22%. This percentage was essentially in line with a previous study, wherein the prevalence of *E. faecalis* was 26% and 32% when identified by culture- and PCR-based methods, respectively (Zandi et al., 2018). The prevalence of *E. faecalis* in primary root canal infections was even lower (7.5%) when investigated using the checkerboard DNA-DNA hybridization (Zahran et al., 2021). These findings collectively support a relatively low occurrence of enterococci in primary endodontic infections. This could be explained by the fact that enterococci are transient members of oral microbiota (Wang et al., 2012), given that endodontic microbiota are derived from oral microbiota influenced by the specific ecological conditions of root canal system (Cogulu et al., 2007). It is also possible that microbial species predominant in primary endodontic infections can inhibit the proliferation of *E. faecalis*, yet such assumption should be investigated in future studies. Our study revealed that *E. faecalis* was identified by 30% in secondary/persistent endodontic infections, which lies in agreement with previous sequencing-based studies, which reported a prevalence of *E. faecalis* equal or greater than 30% in these infections (Siqueira et al., 2002; Zehnder and Belibasakis, 2015). The higher prevalence of *E. faecalis* in secondary/persistent endodontic infections group compared to primary infections in this study (30% vs 22%) agrees with a previous systematic review, which significantly correlated *E. faecalis* with persistent infections (Zhang et al., 2015). Adaptation to environmental conditions of root-filled teeth and tolerance to intracanal disinfection could explain the higher occurrence of *E. faecalis* in post-treatment apical periodontitis (Evans et al., 2002). It has been demonstrated that mechanical instrumentation and exposure to endodontic irrigants increased the number and adhesion forces of *E. faecalis* to dentine and root canal filling materials respectively (Kishen et al., 2008; Vengerfeldt et al., 2014; Keskin et al., 2017). Our study reported a 10% prevalence of *E. faecalis* in saliva samples. Previous studies, both using cultural and molecular methods, reported similar values of prevalence ranging from 19 to 21% (Wang et al., 2012; Delboni et al., 2017). Isolation of *E. faecalis* from saliva samples could be also linked with the isolation of this pathogen from multiple oral sites (Delboni et al., 2017), which supports the assumption that oral cavity could be a potential reservoir of *E. faecalis*. In contrast to our and previous studies, *E. faecalis* was never identified in saliva of patients seeking endodontic retreatment (Zhu et al., 2010). In addition, a significant association was observed between the presence of *E. faecalis* in saliva and root canals, as demonstrated previously (Wang et al., 2012), while contradicting an earlier study (Vidana et al., 2011). The higher odds of identifying *E. faecalis* in root canals when it exists in saliva supports a possible role of *E. faecalis* in saliva as a risk factor for root canal infection with this pathogen. A higher prevalence of *E. faecalis* in saliva and subgingival samples from patients with chronic periodontitis compared to healthy subjects was reported (Xu et al., 2019), and suggests that periodontal infections could favor the colonization of *E. faecalis* as observed in endodontic-periodontal lesions (Guo et al., 2011). For this reason, in our study, subjects with periodontitis were excluded. Our study also agrees with the study by Wang et al. (2012), wherein tooth position, quality and type of restorations were not significantly associated with the presence of *E. faecalis* in root canals despite differences in the demographic characteristics of the investigated populations. Our results demonstrated that the odds of developing a periapical lesion were significantly increased when *E. faecalis* was detected in root canals. These results could be explained by several studies, which demonstrated the role of *E. faecalis* and its virulence factors (such as extracellular proteases and cytolysin) in local inflammation and alveolar bone destruction in apical periodontitis (Souto and Colombo, 2008; Guerreiro-Tanomaru et al., 2013). Our results correlate with the study by Molander et al., wherein enterococci were recovered from 32% of teeth with radiographically verified apical periodontitis versus only 5% in teeth with no apical periodontitis (Molander et al., 1998), while other studies revealed no significant association of enterococci with diseased periapical tissues (Kaufman et al., 2005; Zoletti et al., 2006). Although it is well-established that AP is of bacterial etiology, it is important to consider that multiple local and systemic factors predispose the incidence of periapical lesions and affect the healing of periapical tissues in endodontically-treated teeth (Kirkevang et al., 2007; Holland et al., 2017). Our pilot findings showed that two genetically related salivary isolates of *E. faecalis* were recovered from the same subjects at four months apart, which could support the assumption that this species could persist in the oral cavity for a long time frame, as observed in repeated oral rinses after the ingestion of enterococci-rich food and in mature biofilms recovered from intraoral dental splints (Razavi et al., 2007). We demonstrated genetic relatedness of four pairs of salivary and endodontic *E. faecalis* isolates from the same patient, this supports the hypothesis that *E. faecalis* in saliva could serve as a potential source of infecting root canals. A similar finding was also reported for *E*. *faecalis* strains isolated from saliva, pulp chamber and root canals of endodontic patients (Delboni et al., 2017). These findings can be explained by the possible transition of *E. faecalis* from oral cavity into root canals during or after endodontic treatment or less likely *via* carious lesions. We also found a pair of genetically different *E. faecalis* in saliva and root canals of the same patient (BE51 and BE52). Interestingly, strain BE52 was genetically related to BE5, which was isolated from the root canal of a different patient. These findings suggest that similar strains of *E. faecalis* can be present in different individuals as observed by Pinheiro et al (Pinheiro et al., 2006), which could be related to bacterial intake by exogenous sources such as food (Al-Ahmad et al., 2010). Future studies should be focused on investigating the genetic profiles of *E. faecalis* strains longitudinally collected from the same patient, and their association with food intake. It seems also worthy to explore the long-term occurrence of *E. faecalis* in the oral cavity in a larger cohort, and to investigate the factors which govern the long-term survival of *E. faecalis* and its integration into oral biofilms. The mechanisms which explore the role of *E. faecalis* in the pathogenesis of AP should also be investigated.

## Conclusion

The findings of this study confirmed the presence of *E. faecalis* in saliva and root canals especially those with post-treatment apical periodontitis. The significant association and genetic relatedness of *E*. *faecalis* in saliva and root canals suggest that the presence of *E. faecalis* in saliva is a risk factor for root canal contamination with this pathogen. The latter could increase the risk of developing a periapical lesion. The present study shifts the focus back to the role of *E. faecalis* in the pathogenesis of endodontic infections.

## Data availability statement

The data presented in the study are deposited in the NCBI bioprojects repository, accession number PRJNA891504. https://www.ncbi.nlm.nih.gov/bioproject/?term=PRJNA891504.

## Ethics statement

The studies involving human participants were reviewed and approved by Comitato Etico Regionale per la Sperimentazione Clinica della Regione Toscana Sezione: AREA VASTA SUD EST Segreteria Tecnico Scientifica ubicata c/o: Farmacia Ospedaliera AOUS - Viale Bracci, 16 - 53100 Siena Telefono: 0577-586358 E-mail: c.etico@ao-siena.toscana.it. The patients/participants provided their written informed consent to participate in this study.

## Author contributions

CG: study concept and design, data analysis, writing of original manuscript. CM: performed statistical analysis, study concept and design. IA: data analysis, writing of original manuscript. DP: laboratory experiments, sequencing, genomic and phylogenetic analysis. AF: study concept and sample collection. FS: microbiologic analyses, data analysis, review of manuscript. PN: review of manuscript. GP: study concept, review of manuscript. SG: study concept and design, data analysis, editing and review of manuscript. All authors contributed to the article and approved the submitted version.
